# Spectroscopic and Crystal Field Consequences of Fluoride Binding by [Yb⋅DTMA]^3+^ in Aqueous Solution

**DOI:** 10.1002/anie.201503421

**Published:** 2015-07-27

**Authors:** Octavia A Blackburn, Nicholas F Chilton, Katharina Keller, Claudia E Tait, William K Myers, Eric J L McInnes, Alan M Kenwright, Paul D Beer, Christiane R Timmel, Stephen Faulkner

**Affiliations:** Chemistry Research Laboratory, University of Oxford Mansfield Road, Oxford OX1 3TA (UK) E-mail: Stephen.Faulkner@chem.ox.ac.uk; School of Chemistry, and Photon Science Institute, University of Manchester Oxford Road, Manchester M13 9PL (UK); Centre for Advanced ESR (CÆSR), Inorganic Chemistry Laboratory, University of Oxford South Parks Road, Oxford, OX1 3QR (UK); Department of Chemistry, University of Durham South Road, Durham DH1 3LE (UK)

**Keywords:** anion coordination, lanthanide, magnetic anisotropy, NMR spectroscopy, spectroscopic methods

## Abstract

Yb⋅DTMA forms a ternary complex with fluoride in aqueous solution by displacement of a bound solvent molecule from the lanthanide ion. [Yb⋅DTMA⋅F]^2+^ and [Yb⋅DTMA⋅OH_2_]^3+^ are in slow exchange on the relevant NMR timescale (<2000 s^−1^), and profound differences are observed in their respective NMR and EPR spectra of these species. The observed differences can be explained by drastic modification of the ligand field states due to the fluoride binding. This changes the magnetic anisotropy of the Yb^III^ ground state from easy-axis to easy-plane type, and this change is easily detected in the observed magnetic anisotropy despite thermal population of more than just the ground state. The spectroscopic consequences of such drastic changes to the ligand field represent important new opportunities in developing fluoride-responsive complexes and contrast agents.

Lanthanide complexes have been of interest for many years, partly as a consequence of their applications in time-resolved bioassay, luminescence microscopy and magnetic resonance (MR) imaging.[1] More recently, their magnetic properties have also become the focus of a concerted effort in the area of molecular magnetism.[2] Kinetically stable lanthanide complexes with octadentate ligands derived from tetra-azamacrocycles bearing pendent donor groups have played a key role in all of these areas.

However, while considerable effort has been expended upon lanthanide complexes of DOTA and related tetra-amide ligands, not to mention a host of related structures, there is still much that needs to be understood. Early work focused on the application of the theories developed by Bleaney to understand the NMR spectra of paramagnetic lanthanide complexes with axially symmetric ligand environments. In such systems, the observed chemical shift can be defined as the sum of diamagnetic and paramagnetic contributions, where the paramagnetic contributions, termed the lanthanide induced shift (LIS), can themselves be divided into contact (through bond) and pseudo-contact (through space) terms.[3]

In most lanthanide-containing complexes, *δ_pc_* the pseudo-contact term, is the dominant factor in determining the observed shift for a given nucleus. For an axially symmetric system, this term can be approximated by


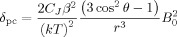
(1)

where *θ* and *r* are the polar coordinates relative to the principal axis, 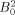 is the second rank axial crystal field parameter, *C_J_*=*g_J_*^2^ *J*(*J*+1)(2 *J*−1)(2 *J*+3)〈*J*|*α*|*J*′〉 is the Bleaney constant and is dependent on the electronic configuration of the lanthanide ion given by the total angular momentum *J*, and *g_J_* is the Landé factor. Parker and co-workers have studied the role of axially coordinated solvent molecules in determining the magnitude of the crystal field parameter,[4] and we have observed that changes to the octadentate donor set from an N4O4 to an N8 donor set can result in dramatic changes to the same parameter.[5] In a different system, Sessler and co-workers observed dramatic changes in ^1^H NMR spectra of lanthanide texaphyrins upon exchange of nitrate with phosphate ligands owing to changes to the crystal field parameters.[6] The simple approximation of Eq. (1) has the advantage of a single crystal field parameter, but necessarily masks the detail of the electronic structure.

Here, we explore the effect of replacing the axial water molecule of [Yb⋅DTMA⋅OH_2_]^3+^ with fluoride as shown in Scheme 1, resulting in remarkable changes to the spectroscopic properties of the complex. Previous work on related systems has been of limited scope; Aime and co-workers commented on fluoride affinity in [Ln⋅(DOTA)]^−^,[7] (Ln=Eu, Yb), though they made no comment about the effect on the electronic structure. Charbonnière, Platas-Iglesias and co-workers have described a system with very high affinity for fluoride in solution, which they ascribed to 2:1 complexation of a single fluoride ion by a pair of lanthanide complexes.[8]

**Scheme 1:**
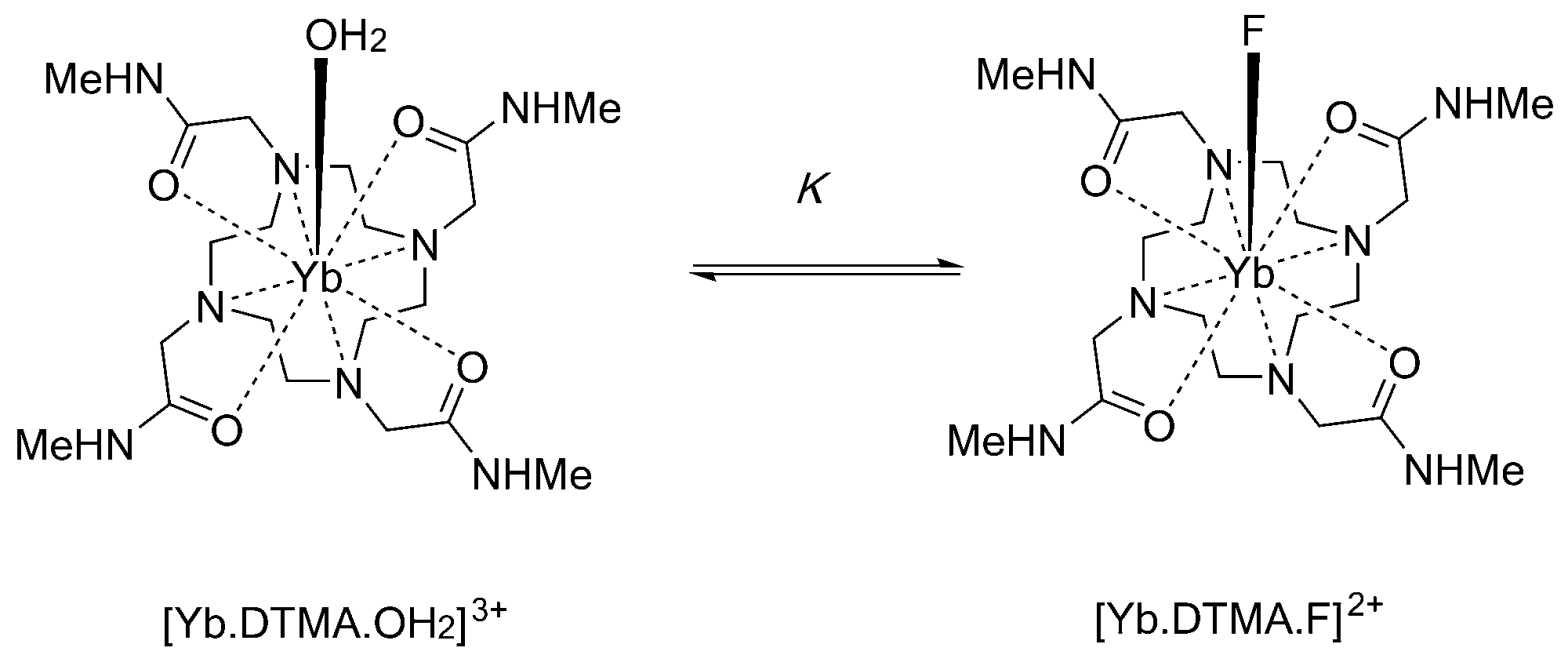
Fluoride binding by [Yb⋅DTMA]^3+^.

[Yb⋅DTMA⋅OH_2_]^3+^ was prepared as its trifluoromethanesulfonate (OTf^−^) salt as described in the literature.[9] Fluoride binding studies were initially investigated by studying the ^1^H NMR spectra of [Yb⋅DTMA⋅OH_2_]^3+^ in D_2_O in the presence and absence of sodium fluoride (Figure 1). It is immediately clear that there are dramatic changes to the observed shifts upon fluoride binding, with a greatly reduced range of shifts compared with the unbound form. Furthermore, the bound and free species are clearly in slow exchange at room temperature, with *k*_ex_<2000 s^−1^, since both sets of resonances are observed in the presence of fluoride. [Yb⋅DTMA⋅OH_2_]^3+^ has capped square antiprismatic (SAP) geometry at the lanthanide center; its spectrum shows no evidence of the alternative diastereoisomers with capped twisted square antiprismatic (TSAP) geometry. The spectrum was assigned on the basis of the published crystal structure of [Dy⋅DTMA⋅OH_2_]^3+^,[9] (Figure S1 in the Supporting Information). Assignment of the resonances observed for [Yb⋅DTMA⋅F]^2+^ was then achieved through EXSY spectroscopy (Figure S2). The EXSY spectrum also reveals that, as well as having a smaller range of observed shifts, the spectrum for [Yb⋅DTMA⋅F]^2+^ is reversed relative to that of [Yb⋅DTMA⋅OH_2_]^3+^, suggesting a dramatic change in the electronic structure of the lanthanide.

**Figure 1:**
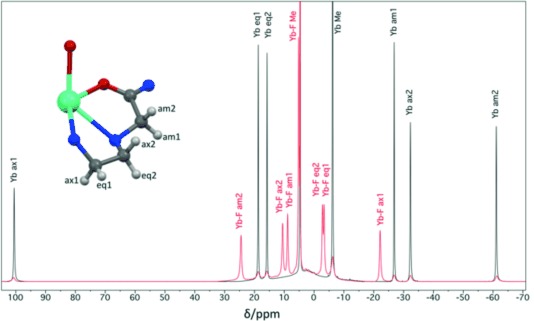
^1^H NMR spectra (400 MHz, 298 K, D_2_O) of [Yb⋅DTMA]^3+^(OTf^−^)_3_ in the absence (black) and presence (red) of an excess of sodium fluoride, with assignments based on the labeled quadrant of the complex as shown.

^19^F NMR spectroscopy confirms that fluoride is bound to the lanthanide in [Yb⋅DTMA⋅F]^2+^; the ^19^F NMR spectrum (Figure S3) shows peaks for free fluoride at −122 ppm and bound fluoride at −858 ppm. Once again, this is consistent with slow exchange on the NMR timescale.

The affinity of [Yb⋅DTMA⋅OH_2_]^3+^ for F^−^ was determined by titration, using ^1^H NMR integrals to quantify the concentrations of the species present. Analysis using Dynafit[6, 11] gave an equilibrium constant of 9.4 m^−1^ with a 95 % confidence interval from 7.6 to 11.4 m^−1^. This value is similar to that determined for a related Eu tetraamide complex, suggesting that it reflects the local donor environment and the residual charge on the lanthanide.[12] Further NMR studies on an analogous yttrium complex, in which a doublet is observed in the ^19^F NMR spectrum,[13] clearly demonstrate that the fluoride complex has 1:1 stoichiometry and that the fluoride is not bridging, rather than adopting the bridging structure found recently by Liu et al. in a related fluoride-containing lanthanide complex.[8]

EPR measurements on solutions of [Yb⋅DTMA⋅OH_2_]^3+^ and [Yb⋅DTMA⋅F]^2+^ in water with 30 % glycerol at 5 K show signals due to the anisotropic ground Kramer’s doublet of the ^2^F_7/2_ term of Yb^III^ (*g_J_*=8/7) in a tetragonal ligand field environment (Figure 2). The native hydrated complex [Yb⋅DTMA⋅OH_2_]^3+^ exhibits an effective *g*-value of *g*_∥_=6.45, with the characteristic hyperfine splitting of *A*_∥_(^171^Yb)=5092 MHz (Figure S6), consistent with the free-ion value scaled by *A_J_*/*g_J_*=*A*/*g*.[14] The asymmetry of the peak at *g*_∥_ suggests a distribution of *g*-values with a mean value of about 6.33, most likely related to structural heterogeneity in frozen solution. The other *g*-values are broadened beyond detection in the frozen solution spectrum, but the EPR spectrum of a 1 % magnetically dilute powder of [Yb⋅DTMA⋅OH_2_]^3+^ in [Lu⋅DTMA⋅OH_2_]^3+^ reveals a further *g*-value of 1.6, in addition to a narrower distribution for the *g*_∥_ signal (Figure S5).

**Figure 2:**
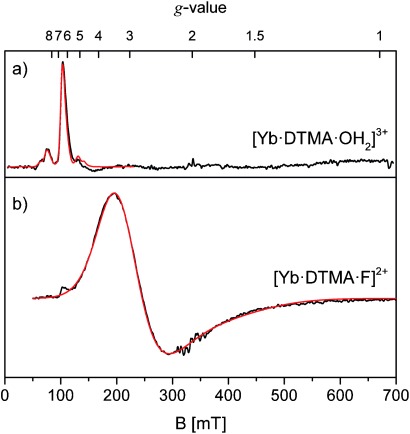
X-band EPR spectra of [Yb⋅DTMA⋅OH_2_]^3+^, 3 mm in 30 % glycerol in water a) in the absence and b) presence of an excess of NaF recorded at 5 and 9 K, respectively (ν_mw_=9.385 GHz, experimental details in the Supporting Information). The corresponding simulations are shown in red for the *g*_∥_ region of [Yb⋅DTMA⋅OH_2_]^3+^ and for the whole spectrum for [Yb⋅DTMA⋅F]^2+^. See Supporting Information for details regarding simulations.

Addition of NaF to [Yb⋅DTMA⋅OH_2_]^3+^ has a pronounced effect on the appearance of the EPR spectrum (Figure 2), leading to a reduction of the *g*-value anisotropy and an inversion from *g*_∥_>*g*_⊥_ for [Yb⋅DTMA⋅OH_2_]^3+^ to *g*_∥_<*g*_⊥_ for [Yb⋅DTMA⋅F]^2+^. By simulation, the *g*-values of [Yb⋅DTMA⋅F]^2+^ were *g*_⊥_=3.00 and *g*_∥_=1.65, with linewidths defined by large *g*-value distributions (*σ*_⊥_=1.20, *σ*_∥_=0.70). Simulations for a distribution of *g*_⊥_ values with a mean value of 2.90, performed in analogy to the simulations for [Yb⋅DTMA⋅OH_2_]^3+^, lead to an improved agreement with experiment. No increased *g*-value resolution could in this case be obtained with the magnetically dilute solid, presumably reflecting a greater inherent microheterogeneity of the ligand coordination sphere in the presence of fluoride. The differences in the *g*-values and *g*-anisotropies between [Yb⋅DTMA⋅OH_2_]^3+^ and [Yb⋅DTMA⋅F]^2+^ reflect a significant change in the electronic structure of the complex, as already suggested by the NMR results.

The solution structures of [Yb⋅DTMA⋅OH_2_]^3+^ and [Yb⋅DTMA⋅F]^2+^ were estimated by gas-phase geometry optimization and symmetrization of their lutetium analogues (see Supporting Information). The DTMA ligand geometry was not found to be significantly different in the two cases. The fluoride ion is calculated to bind with a Ln–F bond length of ca. 1.97 Å. Ab initio calculations for the Yb^3+^ analogues of these pseudo-solution structures (see Supporting Information) give the magnetic susceptibility (*χ*) tensors with the parallel and perpendicular components in Table 2. The calculated average susceptibility values (*χ*_av_) at 300 K are close to the free-ion Yb^3+^ value of 2.57 cm^3^ mol^−1^ K, but lowered 3–6 % due to the ligand field. The most important change on fluoride binding is a change from easy-axis (*χ*_∥_>*χ*_⊥_) to easy-plane (*χ*_∥_<*χ*_⊥_) magnetic anisotropy. This can be understood on the basis that the weak equatorial ligand field of DTMA will stabilize the prolate *m_J_*=±7/2 states of the ^2^F_7/2_ ion; this is consistent with low temperature single crystal magnetic susceptibility studies on Na[Yb⋅DOTA⋅OH_2_]⋅4 H_2_O.[15] Coordination of anionic fluoride on the four-fold axis generates a dominant axial field that stabilizes the oblate *m_J_*=±1/2 states, consistent with the EPR measurements. Pure |*m_J_*|=7/2 and 1/2 Kramer’s doublets would give *g*_∥_, *g*_⊥_=8, 0 and 8/7, 32/7 respectively. The experimental values reflect partial mixing between these two states (Δ*m_J_*=4) as allowed in four-fold symmetry.

The paramagnetic pseudo-contact (dipolar) shifts for each proton were calculated employing Equation (2),[3]


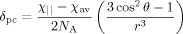
(2)

where *N*_A_ is Avogadro’s number, the susceptibility values are in units of cm^3^ mol^−1^, and *r* is in meters. The calculated ^1^H chemical shifts for [Yb⋅DTMA⋅OH_2_]^3+^ and [Yb⋅DTMA⋅F]^2+^ obtained using the calculated susceptibility values and optimized coordinates are given in Table 1. The contact shift can be neglected in this analysis since such contributions to the LIS are commonly found to be negligible for Yb^3+^ complexes.[16]

**Table 1:** Calculated, observed and fitted pseudo-contact shifts (in ppm) for [Yb⋅DTMA⋅OH_2_]^3+^ and [Yb⋅DTMA⋅F]^2+^

	[Yb⋅DTMA⋅OH_2_]^3+^			[Yb⋅DTMA⋅F]^2+^		
	Calc.^[a]^	Expt.^[b]^	Fit^[c]^	Calc.^[a]^	Expt.^[b]^	Fit^[c]^
H_ax1_	108.7	97.7	93.3	−117.0	−25.0	−26.0
H_eq1_	20.6	15.8	17.7	−26.7	−6.2	−5.9
H_eq2_	17.6	12.9	15.1	−23.7	−5.7	−5.2
H_am1_	−32.4	−29.7	−27.8	32.0	6.0	7.0
H_ax2_	−37.0	−35.2	−31.7	31.9	7.7	7.0
H_am2_	−83.3	−64.0	−71.5	94.9	21.6	21.0

[a] Calculated using Equation (2) and ab initio calculated *χ* values. [b] Assuming a diamagnetic contribution of 2.9 ppm. [c] Fit using Equations (2), (3) and (4).

The calculated shifts for [Yb⋅DTMA⋅OH_2_]^3+^ are clearly in extremely good agreement with the experimental ones, verifying the easy-axis type electronic structure also shown by EPR. The calculated shifts for [Yb⋅DTMA⋅F]^2+^ all have the correct sense, due to the switch in magnetic anisotropy, but they are much larger than the experimental values. As the pseudo-contact shifts are only sensitive to the anisotropy of the susceptibility tensor and not its average, the shifts were then fitted by modifying the *χ* anisotropy while maintaining a constant *χ*_av_ [Eq. (3) and (4)], that is, varying a single parameter (*A*). This approach gives excellent agreement (Table 1) for all ^1^H sites for both complexes, giving confidence in the scaling approach.


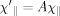
(3)


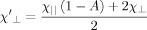
(4)

The fitted *χ* anisotropy is only slightly modified for [Yb⋅DTMA⋅OH_2_]^3+^ (*A*=0.95), also giving confidence in the accuracy of the computational method. Hence, the substantially greater scaling factor required for fitting the shifts of [Yb⋅DTMA⋅F]^2+^ (*A*=2.63, giving a substantially less anisotropic *χ* tensor; Table 2) suggests something more fundamental. Given the good agreement for [Yb⋅DTMA⋅OH_2_]^3+^, the most likely factor is the Yb–F distance taken from the gas-phase optimization.

**Table 2:** Calculated and refined axial magnetic susceptibility components (in cm^3^ mol^−1^ K) at 300 K for [Yb⋅DTMA⋅L]^*n*+^ for L=H_2_O and F^−^

Method	L	*χ*_∥_ *T*	*χ*_⊥_ *T*	*χ*_av_ *T*
ab initio (Yb–F=1.97 Å)	H_2_O	3.8640	1.8147	2.4978
	F^−^	0.7765	3.2141	2.4016
fitting Eq. (3) and (4)	H_2_O	3.6708	1.9113	2.4978
	F^−^	2.0422	2.5813	2.4016
ab initio (Yb–F=2.382 Å)	F^−^	2.1608	2.6952	2.5171

The easy-axis anisotropy of [Yb⋅DTMA⋅OH_2_]^3+^ is transformed to easy-plane type by the destabilizing effect of the negative charge on the axis. If this Yb–F distance was longer in solution than the calculated value, then the disruption would be less and therefore the susceptibility tensor would be expected to be more isotropic. At longer Yb–F distances still, we would expect easy-axis behavior to be recovered. To examine this, ab initio calculations were performed with systematic variation of the Yb–F distance to provide the susceptibility tensors (Figure 3). The anisotropy is very sensitive to the Yb–F distance as expected. Employing polynomial fits of the susceptibility components, the Yb–F bond length required to reproduce the experimental shifts is determined as 2.382 Å. This is substantially longer than the gas-phase optimized length of 1.97 Å, but is still less than that for a bound water molecule. Preliminary DFT optimizations show that the optimized Lu–F bond length substantially increases in the presence of explicit water molecules and a solvent continuum model (see Supporting Information), suggesting that it is hydrogen bonding interactions between the bound fluoride and solvent that is responsible for this discrepancy.

**Figure 3:**
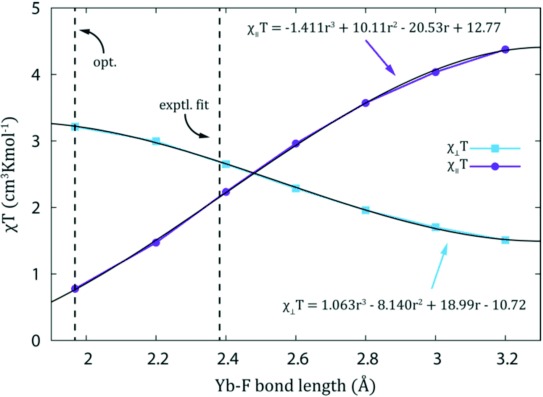
Ab initio calculated magnetic susceptibility tensors of [Yb⋅DTMA⋅F]^2+^ as a function of Yb–F bond length.

In conclusion, it is particularly worthy of note that the electronic structure of the ytterbium center in [Yb⋅DTMA]^3+^, and therefore its characteristic magnetic anisotropy, can be modified from easy-axis to easy-plane by replacement of water with a fluoride ion. Variation of the crystal field has already been shown to be important for molecular magnetism, and our observation that changing a single donor can radically alter the spectroscopic properties of a complex raises the prospect of achieving new levels of control and understanding. There are also clear implications for developing sensors and responsive probes for fluoride. In particular, the possibilities for CEST (chemical exchange saturation transfer) imaging of fluoride are clearly very good, and we are currently exploring the potential of both ^1^H and ^19^F NMR methods.

## Supporting Information

As a service to our authors and readers, this journal provides supporting information supplied by the authors. Such materials are peer reviewed and may be re-organized for online delivery, but are not copy-edited or typeset. Technical support issues arising from supporting information (other than missing files) should be addressed to the authors.

miscellaneous_information
