# Supplementary material for: Spectroscopic and Crystal Field Consequences of Fluoride Binding by [Yb⋅DTMA]3+ in Aqueous Solution
Source: Angew Chem Int Ed Engl. 2015 Jul 27;54(37):10783–6. doi: 10.1002/anie.201503421 (PMC4611957; doi:10.1002/anie.201503421)
Supplement: Supplementary file 1 — miscellaneous_information [file anie0054-10783-sd1.pdf]

Angewandte Chemie
Eine Zeitschrift der Gesellschaft Deutscher Chemiker

Supporting Information

Spectroscopic and Crystal Field Consequences of Fluoride Binding by $[Yb{\cdot}DTMA]^{3+}$ in Aqueous Solution

*Octavia A. Blackburn, Nicholas F. Chilton, Katharina Keller, Claudia E. Tait, William K. Myers, Eric J. L. McInnes, Alan M. Kenwright, Paul D. Beer, Christiane R. Timmel, and Stephen Faulkner**

anie_201503421_sm_miscellaneous_information.pdf

Supplementary Information:

Contents:

1 **Materials and Methods** 1
2 **^{1}H and ^{19}F NMR** 2
3 **Equilibrium constant** 4
4 **EPR Spectroscopy** 5
5 **Computational details** 7
6 **Computational results** 8
7 **References** 17

1 Materials and Methods

The ligand DTMA (1,4,7,10-tetrakis[(*N*-methylcarbamoyl)methyl]-1,4,7,10-tetraaza-cyclododecane) and its lanthanide complex $YbDTMA(OTf)_3$ was synthesised as previously described.[1] The complex was characterised by NMR, mass spectrometry and CHN analysis.

NMR measurements were made using either a Bruker AVIII-500 (^{1}H = 500 MHz, ^{19}F = 470 MHz) or a Bruker AVIII-400 (^{1}H = 400 MHz, ^{19}F = 376 MHz). Wide spectral width spectra were obtained using pulses sufficiently short to ensure reasonably uniform excitation over the observed bandwidth, and with acquisition and recycle times matched to the relaxation characteristics of the lanthanide complexes (repetition time typically 100 ms). EXSY spectra were recorded at 500 MHz using a gradient selected NOESY sequence with a mixing time of 1 ms.

To obtain an equilibrium constant by ^{1}H NMR, 0.005M YbDTMA was titrated with 0.8M NaF in D_2O at 298 K. A non-dilution method was used where 0.5 mL of the YbDTMA stock solution was put in an NMR tube and a solution of NaF made with the same stock solution was titrated in using a glass microliter syringe. The water-soluble standard DSS (4,4-dimethyl-4-silapentane-1-sulfonic acid) was included to provide signals to integrate against. The delay time (d1) used was at least 5 times longer than the T1 of the slowest relaxing peak in order to make integration quantitative. Spectra were individually phased and baseline corrected before analysis.

The increasing signals of the fluoride-bound species and the decreasing signals of the original species were both monitored and averaged respectively to give two independent data sets. These were then fitted simultaneously in Dynafit[2] to give the association constant K using the equilibrium:

$$(LnDTMA - H_2O)^{3+} + F^- \rightleftharpoons (LnDTMA - F)^{2+} + H_2O$$

$$K = \frac{[(LnDTMA - F)^{2+}]}{[(LnDTMA - H_2O)^{3+}] + [F^-]}$$

The following script was used in Dynafit:

```
[task]
data = equil
task = fit
[mechanism]
Yb + F <==> complex1 : K1 assoc
[constants]
K1 = 10 ??
[concentrations]
Yb = 0.005
[responses]
[equil]
variable F
file ./NMR/YbDTMA_F_NMR_1H_new.txt |response complex1 = 60?
file ./NMR/YbDTMA_F_NMR_1H_original.txt |response Yb = 106?
[output]
directory ./output/NMR/YbDTMA_F_1H
[end]
```

2 ^{1}H and ^{19}F NMR

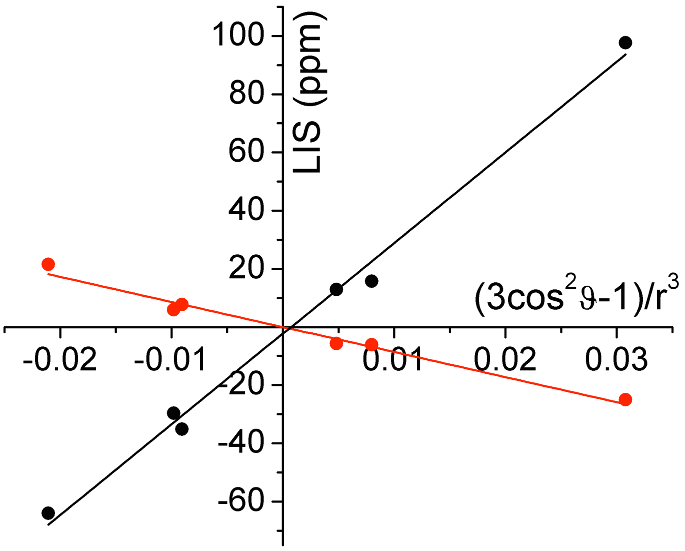

Figure S1: Bleaney plots for YbDTMA proton resonances in the absence (black) and presence (red) of an excess of sodium fluoride in D_2O, using atomic coordinates from the crystal structure of DyDTMA[1]

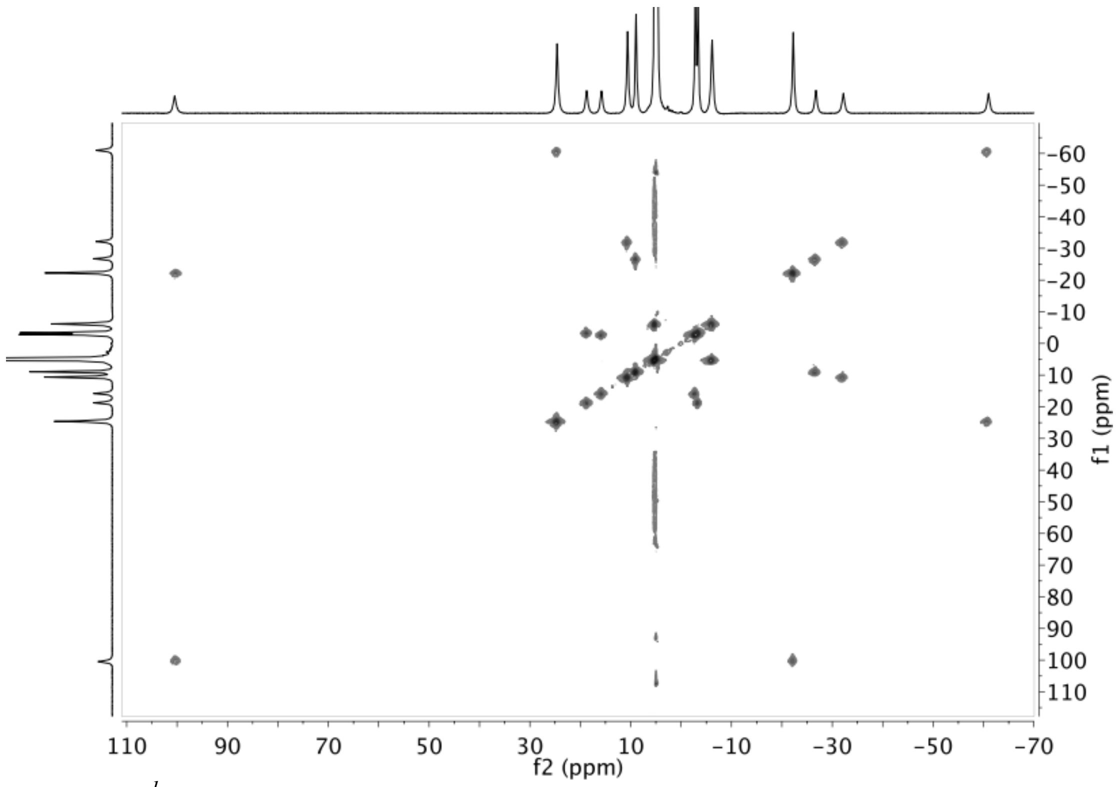

Figure S2. ^1H EXSY spectrum of YbDTMA with added fluoride at 298 K with a mixing time of 1 ms at 500 MHz.

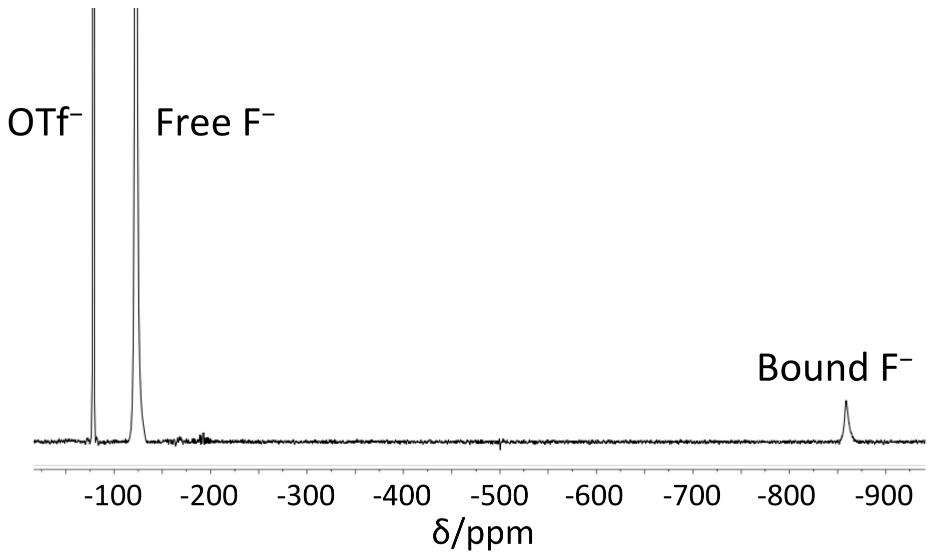

Figure S3: ^{19}F NMR spectrum (470 MHz, 298 K, D_2O) of the trifluoromethanesulfonate salt of YbDTMA in the presence of excess fluoride.

3 Equilibrium constant

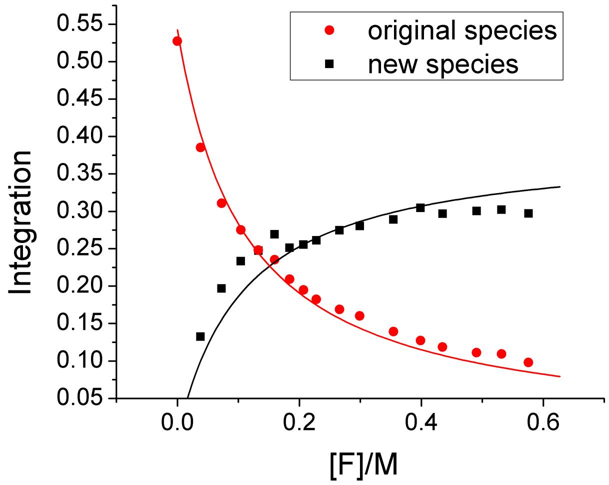

Figure S4. Binding isotherms and fits for titration of YbDTMA with NaF monitoring ^{1}H NMR integrals relative to DSS.

4 EPR Spectroscopy[3]

Samples for the EPR measurements were thoroughly degassed with at least three freeze-pump-thaw cycles and flame-sealed under vacuum.
X-band CW-EPR data was collected on a Bruker BioSpin Premium EMX^{micro} spectrometer and a Bruker BioSpin ElexSys E680 spectrometer with a Bruker SHQE-W TE_{011} cylindrical mode cavity and Oxford Instruments ESR-900 cryostat. Data were acquired with a modulation amplitude of 8 G, time constant of 81.92 ms, sweep time of 2 min. The microwave power was adjusted so as to prevent saturation for each acquisition. Additional measurements at S- and Q-band, at the EPSRC National EPR Facility at the University of Manchester on Bruker Elexsys E580 and EMX spectrometers, were performed to confirm the effective g-values.

EPR simulations were performed in MATLAB™ with the EasySpin toolbox.[4] The sample temperature was included as a parameter in the simulations. The g-values were fitted to the experimental spectrum and the Yb hyperfine couplings used in the simulations were calculated from the g-value for the corresponding orientation according to the relationship $A_i(^{171}Yb)$ = [796 ± 2.5 MHz] g_i-[43 ± 3 MHz]. This equation was determined based on literature data on Yb^{3+} in different compounds[5] and is valid if the ground state is derived exclusively from the $^2F_{7/2}$ free ion state. Additional measurements at S- and Q-band, at the EPSRC National EPR Facility at the University of Manchester on Bruker Elexsys E580 and EMX spectrometers, were performed to confirm the effective g-values.

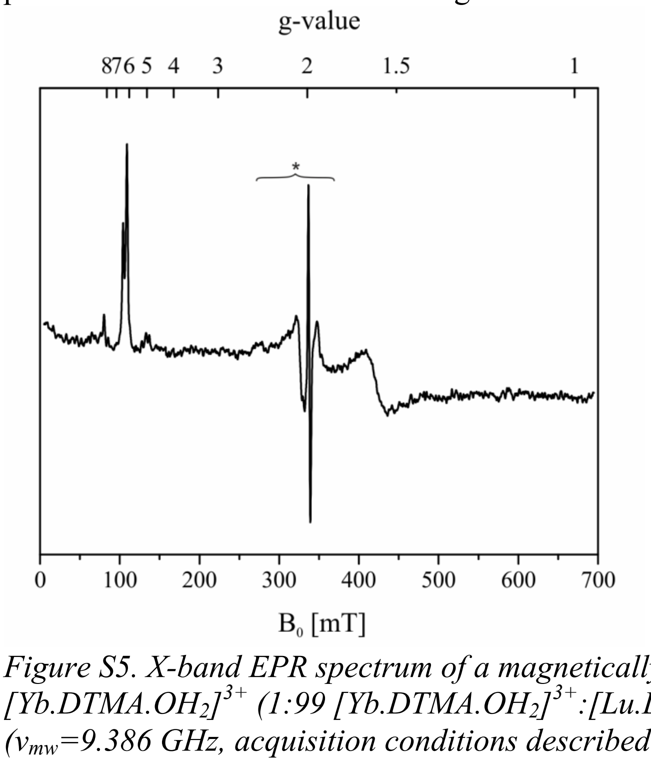

Figure S5. X-band EPR spectrum of a magnetically dilute powder of $[Yb.DTMA.OH_2]^{3+}$ (1:99 $[Yb.DTMA.OH_2]^{3+}$:$[Lu.DTMA.OH_2]^{3+}$) recorded at 5 K (ν_{mw}=9.386 GHz, acquisition conditions described in the text). The asterisk indicates a region of background signals.

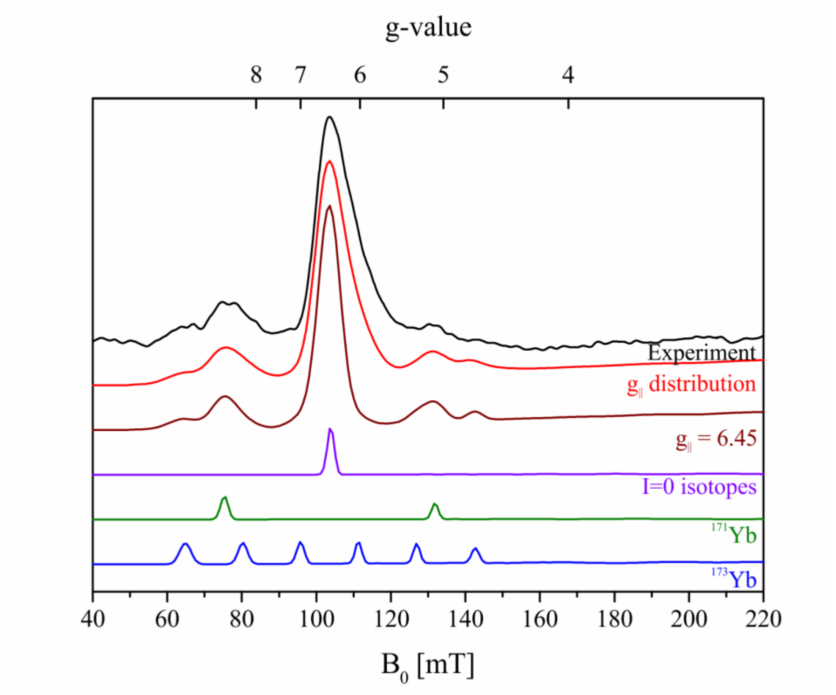

*Figure S6. X-band EPR simulations of the $g_{||}$ region of the spectrum of $[Yb.DTMA.OH_2]^{3+}$ recorded in frozen solution at 5 K (ν_{mw}=9.386 GHz). The parameters for the simulation shown in dark red were $g_{||}$=6.45, A(^{173}Yb)=1401 MHz, and A(^{171}Yb)=5092 MHz, with linewidths defined by g-strain (0.4 FWHM) and A-strain (75 MHz FWHM). The contributions of different Yb isotopes are shown below (not to scale). The simulation in red was performed by considering a distribution of g-values based on the slope on the high-field side of the $g_{||}$=6.45 peak. The best fit was obtained by summing spectra with g-values in the range from 6.6-5.6 with a scaling factor determined by the amplitude Y at the field position X corresponding to that g-value calculated according to Y(EPR a.u.)=-0.0530 (EPR a.u.) *X(mT)+6.4814 (EPR a.u.). The origin of the structure of the g = 6.3 feature observed in the neat powder is not clear but the splitting is field independent, i.e. it behaves as a coupling rather than as due to different effective g-values. We speculate that it is inter-molecular in nature: the splitting is not observed in the magnetically dilute material.*

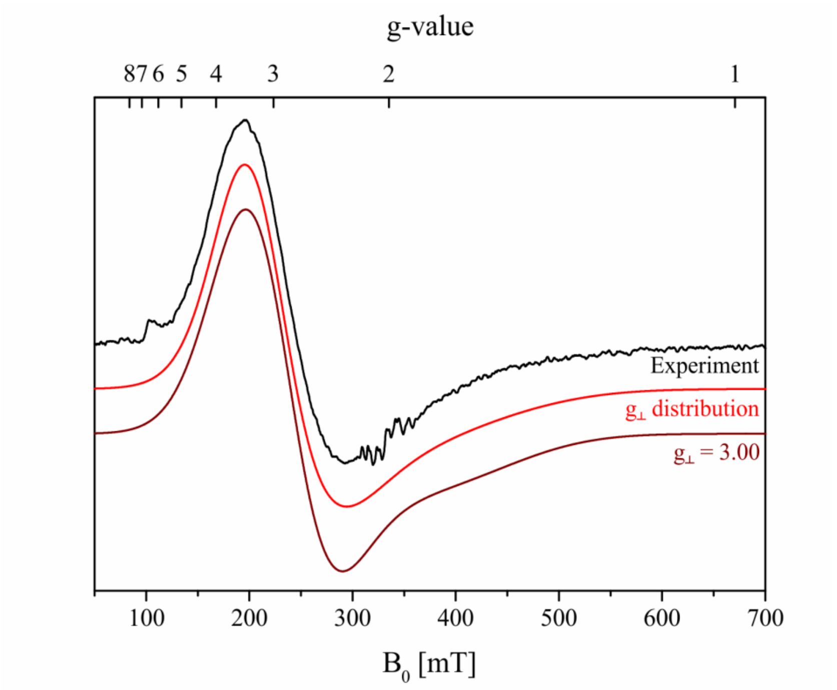

*Figure S7. X-band EPR simulations of the spectrum of [Yb.DTMA.F]$^{2+}$ recorded in frozen solution at 9 K (ν_{mw}=9.384 GHz). The parameters for the simulation shown in dark red were $g_\perp$=3.00, $g_{||}$=1.65, [$A_\perp$ $A_{||}$](^{173}Yb)=[645 350] MHz, and [$A_\perp$ $A_{||}$](^{171}Yb)=[2345 1270] MHz, with linewidths defined by g-strain (1.2 and 0.7 FWHM) and A-strain (250 and 140 MHz FWHM). The simulation in red was performed by considering a distribution of $g_\perp$-values in analogy to the simulation in Figure S6. The best fit was obtained with g-values in the range from 3.2-2.5 and scaling by an amplitude Y at the corresponding field position X calculated according to Y(EPR a.u.)= -0.0182 (EPR a.u.) *X(mT)+ 5.4545 (EPR a.u.).*

5 Computational details

Compound geometries were optimized with Density Functional Theory (DFT) using the B3LYP functional[6] with DFTD3 dispersion corrections,[7] the RIJCOSX approximation,[8] the ZORA relativistic treatment[9] and the SARC-def2-TZVP basis[10] as implemented in ORCA 3.0.2.[11] The Yb^{3+} ions were replaced by the diamagnetic Lu^{3+} ions to simplify the DFT convergence. The DTMA geometries were then symmetrized yielding four-fold symmetric structures. *Ab initio* calculations were performed using the CASSCF/RASSI/SINGLE_ANISO approach,[12] using MOLCAS 7.8.[13] For all calculations the Yb atom was treated with the ANO-RCC-VTZP basis, the N, O and F donors with the ANO-RCC-VDZP basis, while all other atoms were treated with treated with the ANO-RCC-VDZ basis.[14] The two electron integrals were Cholesky decomposed with the default thresholds. The $4f^{13}$ configuration of Yb^{III} was modelled with a complete active space of 13 electrons in 7 orbitals where the 7 doublets were included in the orbital optimization and then mixed by spin-orbit coupling. Simulation of the EPR spectra for the crystal field multiplets was performed with PHI,[15] using the crystal field decomposition from SINGLE_ANISO.

6 Computational results

Table S1. DFT optimized and symmetrized coordinates of [Lu.DTMA.OH$_2$]$^{3+}$

	x	y	z
Lu1	0	0	0
O1w	0	0	2.398027876
H1w	-0.136594063	-0.7958725	2.924992693
H1w	-0.278583774	0.75939963	2.922470102
O1	0.138070524	-2.180069999	0.698816873
N1	-1.229000146	-1.71562694	-1.623443969
N2	-0.374634839	-4.382966987	0.648523681
C1	1.104718802	-2.435256729	-2.236652267
C2	-0.317941831	-2.183522643	-2.699423239
C3	-1.628640172	-2.836475403	-0.752641969
C4	-0.543450318	-3.134549918	0.258305813
C5	0.598460957	-4.819740111	1.650691056
H1n	-0.954168074	-5.093622615	0.224664698
H2a	1.133598873	-3.240158384	-1.507443352
H2e	1.695059181	-2.780593442	-3.091942902
H1a	-0.318170102	-1.428919722	-3.482399186
H1e	-0.710338899	-3.097589191	-3.159120824
H1am	-1.905304327	-3.724211139	-1.330570444
H2am	-2.498899561	-2.528579719	-0.172300726
H1m	1.423625147	-5.339355466	1.164030634
H1m	0.116512298	-5.498092797	2.350874942
H1m	0.972750164	-3.951694749	2.183051341
O1	2.180069999	0.138070524	0.698816873
N1	1.71562694	-1.229000146	-1.623443969
N2	4.382966987	-0.374634839	0.648523681
C1	2.435256729	1.104718802	-2.236652267
C2	2.183522643	-0.317941831	-2.699423239
C3	2.836475403	-1.628640172	-0.752641969
C4	3.134549918	-0.543450318	0.258305813
C5	4.819740111	0.598460957	1.650691056
H1n	5.093622615	-0.954168074	0.224664698
H2a	3.240158384	1.133598873	-1.507443352
H2e	2.780593442	1.695059181	-3.091942902
H1a	1.428919722	-0.318170102	-3.482399186
H1e	3.097589191	-0.710338899	-3.159120824
H1am	3.724211139	-1.905304327	-1.330570444
H2am	2.528579719	-2.498899561	-0.172300726
H1m	5.339355466	1.423625147	1.164030634
H1m	5.498092797	0.116512298	2.350874942
H1m	3.951694749	0.972750164	2.183051341
O1	-0.138070524	2.180069999	0.698816873
N1	1.229000146	1.71562694	-1.623443969
N2	0.374634839	4.382966987	0.648523681
C1	-1.104718802	2.435256729	-2.236652267
C2	0.317941831	2.183522643	-2.699423239
C3	1.628640172	2.836475403	-0.752641969
C4	0.543450318	3.134549918	0.258305813
C5	-0.598460957	4.819740111	1.650691056
H1n	0.954168074	5.093622615	0.224664698
H2a	-1.133598873	3.240158384	-1.507443352
H2e	-1.695059181	2.780593442	-3.091942902
H1a	0.318170102	1.428919722	-3.482399186

H1e	0.710338899	3.097589191	-3.159120824
H1am	1.905304327	3.724211139	-1.330570444
H2am	2.498899561	2.528579719	-0.172300726
H1m	-1.423625147	5.339355466	1.164030634
H1m	-0.116512298	5.498092797	2.350874942
H1m	-0.972750164	3.951694749	2.183051341
O1	-2.180069999	-0.138070524	0.698816873
N1	-1.71562694	1.229000146	-1.623443969
N2	-4.382966987	0.374634839	0.648523681
C1	-2.435256729	-1.104718802	-2.236652267
C2	-2.183522643	0.317941831	-2.699423239
C3	-2.836475403	1.628640172	-0.752641969
C4	-3.134549918	0.543450318	0.258305813
C5	-4.819740111	-0.598460957	1.650691056
H1n	-5.093622615	0.954168074	0.224664698
H2a	-3.240158384	-1.133598873	-1.507443352
H2e	-2.780593442	-1.695059181	-3.091942902
H1a	-1.428919722	0.318170102	-3.482399186
H1e	-3.097589191	0.710338899	-3.159120824
H1am	-3.724211139	1.905304327	-1.330570444
H2am	-2.528579719	2.498899561	-0.172300726
H1m	-5.339355466	-1.423625147	1.164030634
H1m	-5.498092797	-0.116512298	2.350874942
H1m	-3.951694749	-0.972750164	2.183051341

Table S2. DFT optimized and symmetrized coordinates of $[Lu.DTMA.F]^{2+}$

	**x**	**y**	**z**
Lu1	0	0	0
F1	0	0	1.967922391
O1	-0.137896936	2.302591721	0.431487524
N1	1.290410773	1.703994917	-1.826889258
N2	0.56794775	4.442064039	0.4272012
C1	-1.044569751	2.464012213	-2.437279751
C2	0.377958983	2.173581356	-2.890167156
C3	1.72435035	2.810868004	-0.967247012
C4	0.637105683	3.180036822	0.021387334
C5	-0.3871255	4.910741845	1.42925235
H1n	1.209386765	5.109639833	0.028123386
H2a	-1.055977547	3.275290088	-1.715616117
H2e	-1.609972651	2.823979517	-3.306197355
H1a	0.356555862	1.409616586	-3.664076418
H1e	0.781449598	3.076498573	-3.367900021
H1am	2.051121947	3.682079764	-1.548967407
H2am	2.567964657	2.468182812	-0.367122258
H1m	-1.246325806	5.380061081	0.947187853
H1m	0.099142297	5.637833109	2.076134121
H1m	-0.718202815	4.062453943	2.02154114
O1	-2.302591721	-0.137896936	0.431487524
N1	-1.703994917	1.290410773	-1.826889258
N2	-4.442064039	0.56794775	0.4272012
C1	-2.464012213	-1.044569751	-2.437279751
C2	-2.173581356	0.377958983	-2.890167156
C3	-2.810868004	1.72435035	-0.967247012
C4	-3.180036822	0.637105683	0.021387334
C5	-4.910741845	-0.3871255	1.42925235
H1n	-5.109639833	1.209386765	0.028123386

H2a	-3.275290088	-1.055977547	-1.715616117
H2e	-2.823979517	-1.609972651	-3.306197355
H1a	-1.409616586	0.356555862	-3.664076418
H1e	-3.076498573	0.781449598	-3.367900021
H1am	-3.682079764	2.051121947	-1.548967407
H2am	-2.468182812	2.567964657	-0.367122258
H1m	-5.380061081	-1.246325806	0.947187853
H1m	-5.637833109	0.099142297	2.076134121
H1m	-4.062453943	-0.718202815	2.02154114
O1	0.137896936	-2.302591721	0.431487524
N1	-1.290410773	-1.703994917	-1.826889258
N2	-0.56794775	-4.442064039	0.4272012
C1	1.044569751	-2.464012213	-2.437279751
C2	-0.377958983	-2.173581356	-2.890167156
C3	-1.72435035	-2.810868004	-0.967247012
C4	-0.637105683	-3.180036822	0.021387334
C5	0.3871255	-4.910741845	1.42925235
H1n	-1.209386765	-5.109639833	0.028123386
H2a	1.055977547	-3.275290088	-1.715616117
H2e	1.609972651	-2.823979517	-3.306197355
H1a	-0.356555862	-1.409616586	-3.664076418
H1e	-0.781449598	-3.076498573	-3.367900021
H1am	-2.051121947	-3.682079764	-1.548967407
H2am	-2.567964657	-2.468182812	-0.367122258
H1m	1.246325806	-5.380061081	0.947187853
H1m	-0.099142297	-5.637833109	2.076134121
H1m	0.718202815	-4.062453943	2.02154114
O1	2.302591721	0.137896936	0.431487524
N1	1.703994917	-1.290410773	-1.826889258
N2	4.442064039	-0.56794775	0.4272012
C1	2.464012213	1.044569751	-2.437279751
C2	2.173581356	-0.377958983	-2.890167156
C3	2.810868004	-1.72435035	-0.967247012
C4	3.180036822	-0.637105683	0.021387334
C5	4.910741845	0.3871255	1.42925235
H1n	5.109639833	-1.209386765	0.028123386
H2a	3.275290088	1.055977547	-1.715616117
H2e	2.823979517	1.609972651	-3.306197355
H1a	1.409616586	-0.356555862	-3.664076418
H1e	3.076498573	-0.781449598	-3.367900021
H1am	3.682079764	-2.051121947	-1.548967407
H2am	2.468182812	-2.567964657	-0.367122258
H1m	5.380061081	1.246325806	0.947187853
H1m	5.637833109	-0.099142297	2.076134121
H1m	4.062453943	0.718202815	2.02154114

Table S3. Kramers doublets of the $^2F_{7/2}$ multiplet of Yb^{III} in the optimized and symmetrized $[Yb.DTMA.OH_2]^{3+}$. Lack of pure axial symmetry is due to the fixed position of the water molecule which would of course be freely rotating in solution.

Energy (cm^{-1})	g_x	g_y	g_z
0	0.03	0.61	7.39
160	2.74	2.31	4.56
329	5.20	2.65	1.22
361	6.65	1.42	0.49

Table S4. Kramers doublets of the $^2F_{7/2}$ multiplet of Yb^{III} in the optimized and symmetrized $[Yb.DTMA.F]^{2+}$, where the Yb-F bond length is 1.97 Å.

Energy (cm^{-1})	g_x	g_y	g_z
0	4.47	4.40	0.92
222	1.59	1.66	3.05
522	1.76	1.50	5.30
614	0.25	0.00	7.73

Table S5. Kramers doublets of the $^2F_{7/2}$ multiplet of Yb^{III} in the optimized and symmetrized $[Yb.DTMA.F]^{2+}$, where the Yb-F bond length is 2.382 Å.

Energy (cm^{-1})	g_x	g_y	g_z
0	3.02	3.24	1.72
73	3.34	3.10	1.51
207	2.51	0.06	4.80
220	4.30	1.84	3.46

The first excited crystal field doublets lie at approximately 160 and 73 cm^{-1}, for $[Yb.DTMA.OH_2]^{3+}$ and $[Yb.DTMA.F]^{2+}$ respectively (see Tables S3 and S5), and

therefore no transitions to these doublets would be expected at X-band (~0.3 cm^{-1}). Furthermore, at the cryogenic temperatures at which the EPR spectra were collected, 5 and 9 K respectively, there is no population (< 0.0001 %) in the first excited doublets and therefore we do not observe any transitions from within these excited states. The Zeeman splitting (x and z orientations) and calculated EPR spectra based on the entire crystal field multiplet as determined through *ab initio* are shown below. These clearly show the same features as the experimental spectra, highlighting that the transitions arise from the ground Kramers doublet.

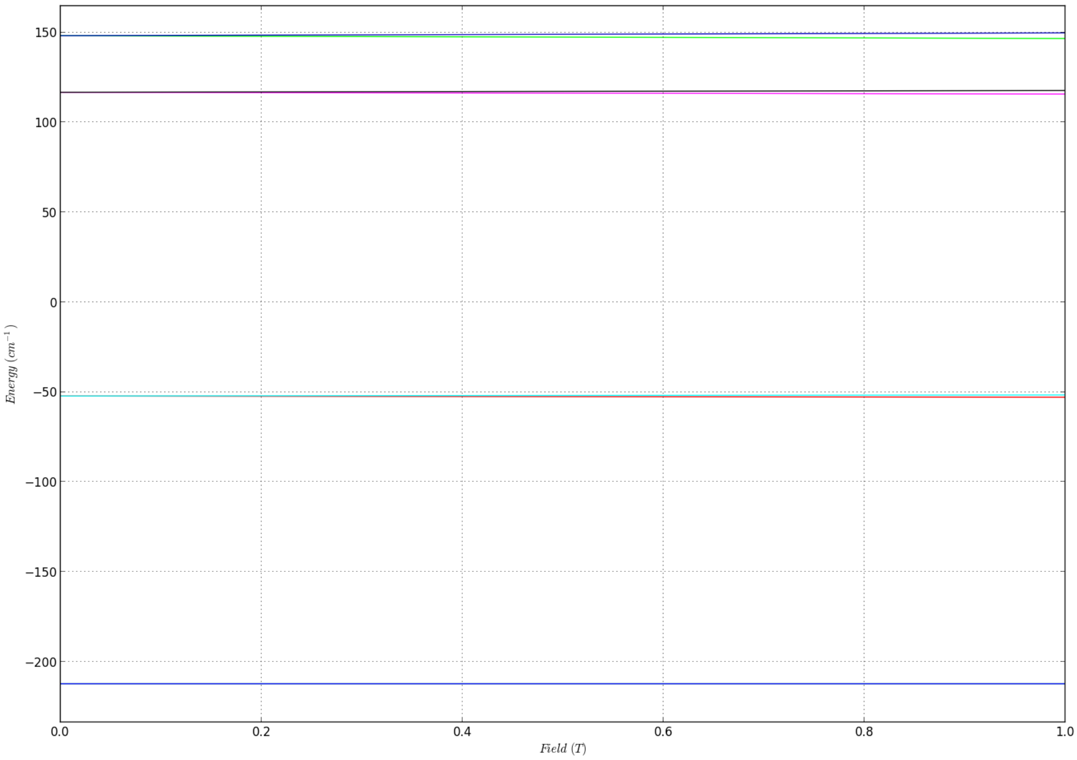

Figure S8. Zeeman splitting of the $^2F_{7/2}$ multiplet of Yb^{III} with the external field along the x-axis, in the optimized and symmetrized $[Yb.DTMA.OH_2]^{3+}$.

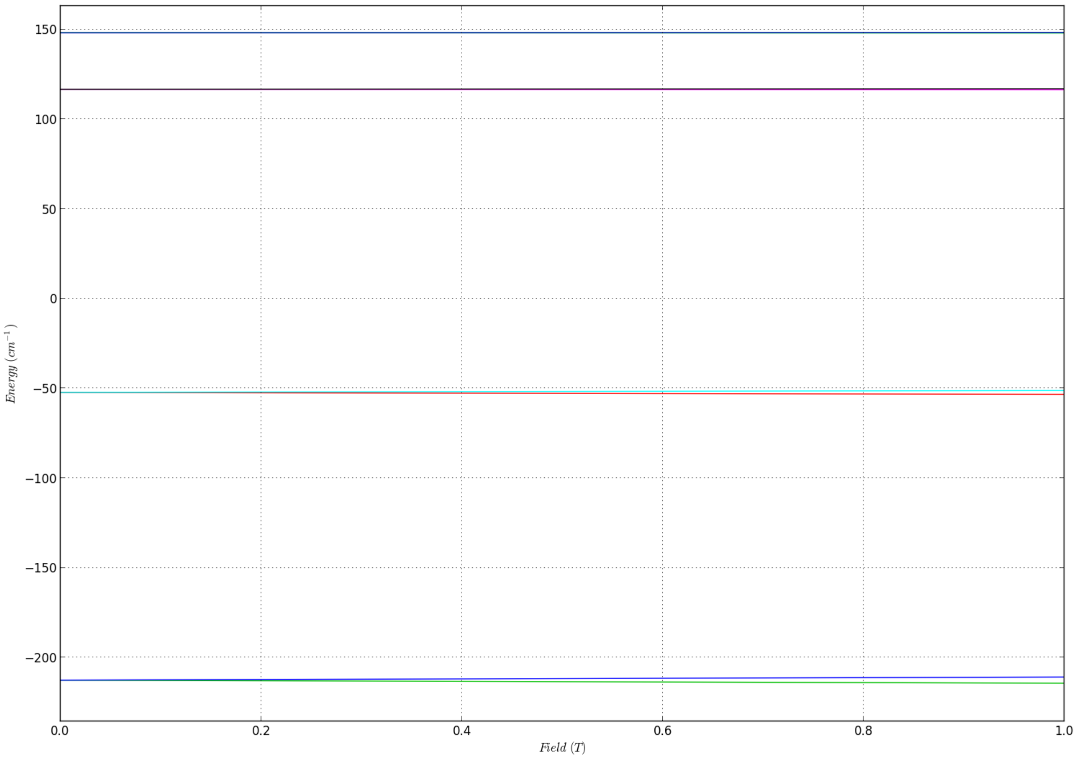

Figure S9. Zeeman splitting of the $^2F_{7/2}$ multiplet of Yb^{III} with the external field along the z-axis, in the optimized and symmetrized $[Yb.DTMA.OH_2]^{3+}$.

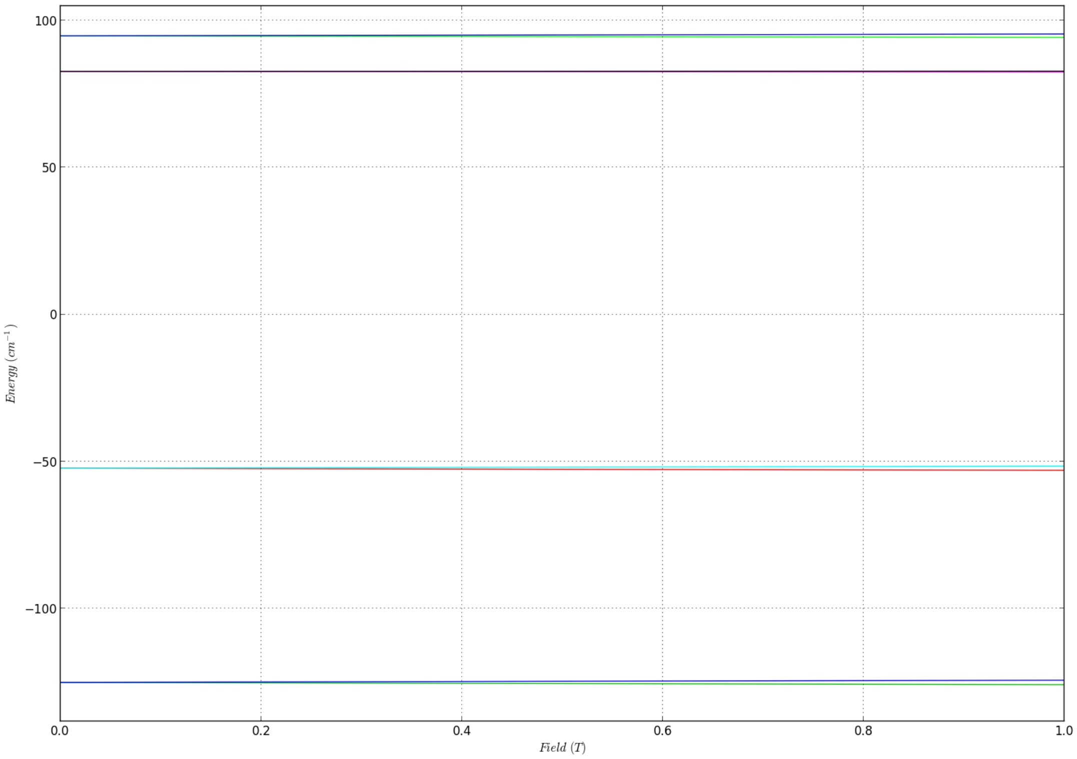

Figure S10. Zeeman splitting of the $^2F_{7/2}$ multiplet of Yb^{III} with the external field along the x-axis, in the optimized and symmetrized $[Yb.DTMA.F]^{2+}$, where the Yb-F bond length is 2.382 Å.

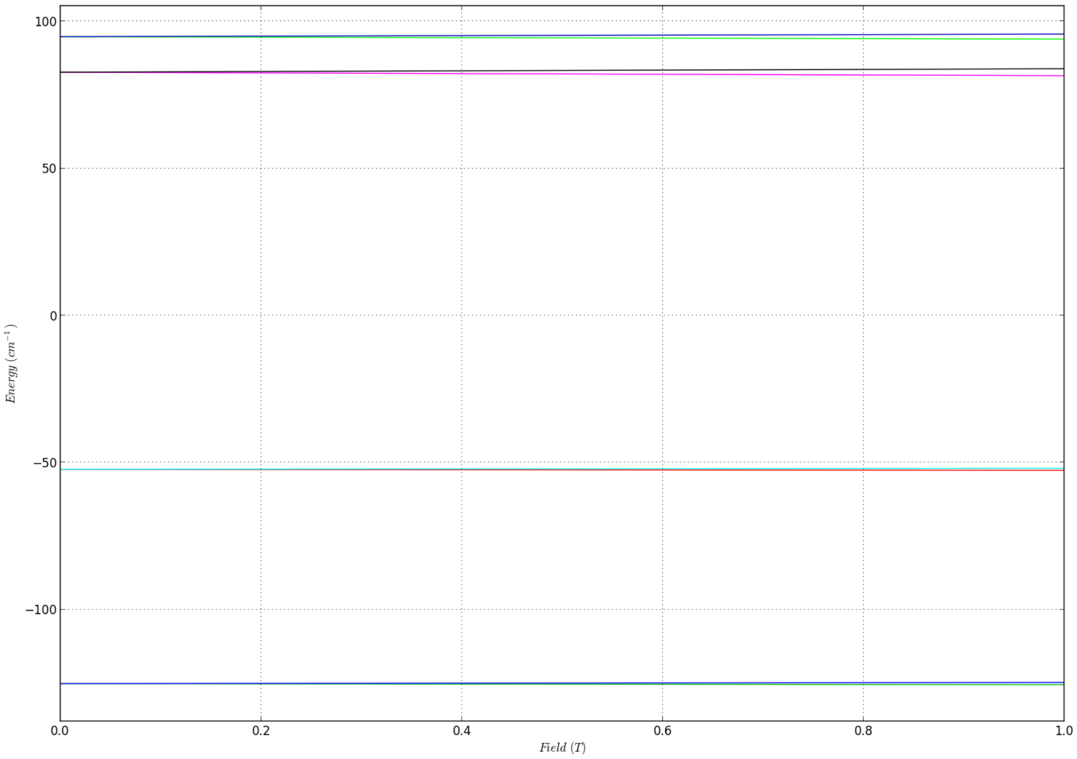

Figure S11. Zeeman splitting of the $^2F_{7/2}$ multiplet of Yb^{III} with the external field along the z-axis, in the optimized and symmetrized $[Yb.DTMA.F]^{2+}$, where the Yb-F bond length is 2.382 Å.

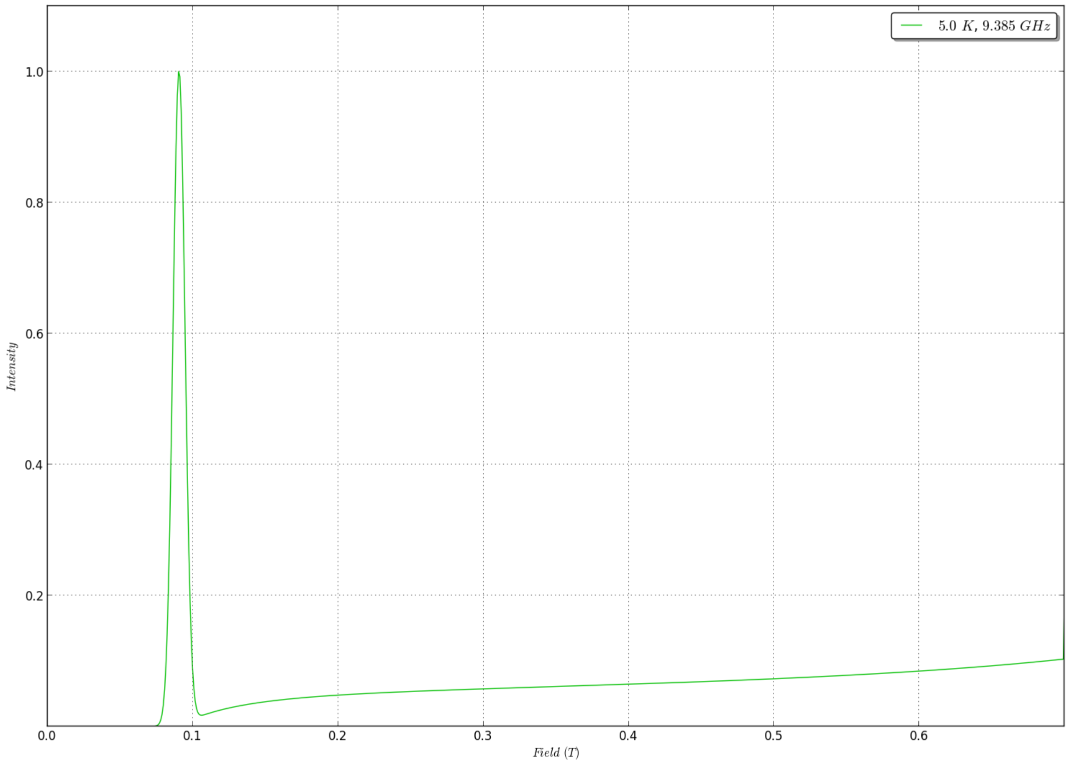

Figure S12. Calculated X-band EPR spectra for the optimized and symmetrized $[Yb.DTMA.OH_2]^{3+}$, employing the entire $^2F_{7/2}$ crystal field multiplet.

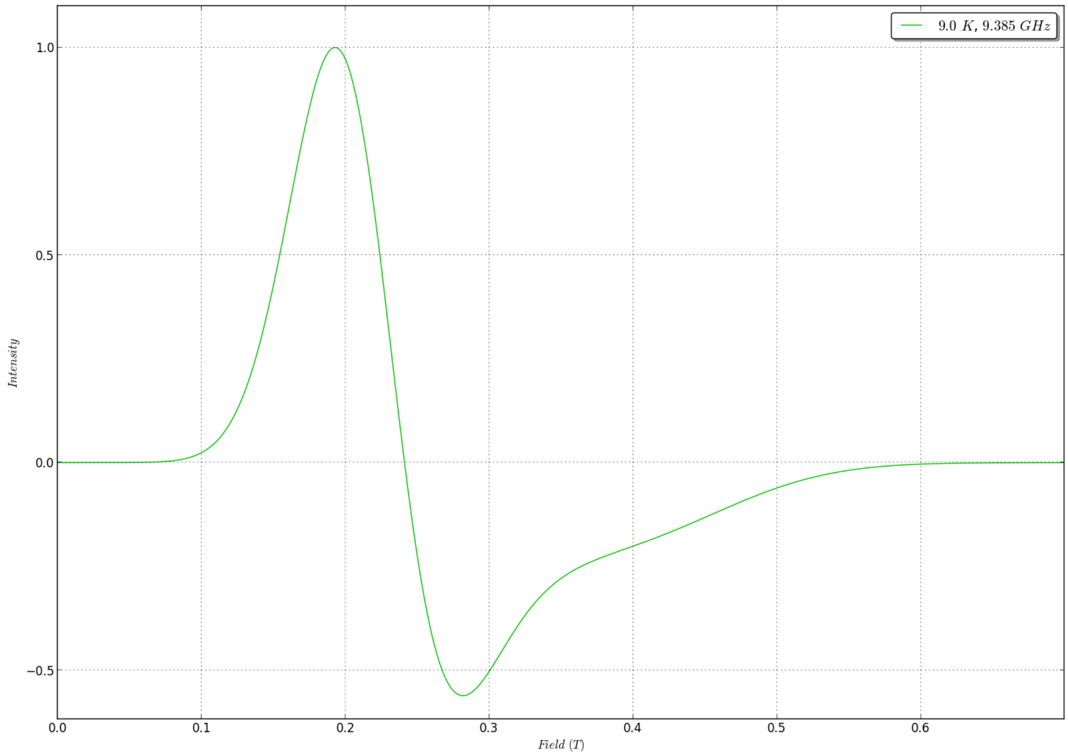

Figure S13. Calculated X-band EPR spectra for the optimized and symmetrized $[Yb.DTMA.F]^{2+}$, where the Yb-F bond length is 2.382 Å, employing the entire $^{2}F_{7/2}$ crystal field multiplet.

Table S6. DFT optimized coordinates of $[Lu.DTMA.F]^{2+}.7H_2O$, in the presence of a water solvent continuum, using the COSMO approach.

	**x**	**y**	**z**
Lu1	0	0	0
F1	0	0	2.129540432
O1	1.534744927	1.607294898	0.685295915
N1	2.148438425	0.201768693	-1.570376388
N2	3.545397271	2.627602775	0.689560628
C1	1.213409568	2.445751922	-2.200439032
C2	1.94333023	1.197059441	-2.647235727
C3	3.227857235	0.63227695	-0.666944593
C4	2.716369322	1.679104073	0.292785421
C5	3.184355636	3.667779917	1.64322033
H1n	4.47280993	2.644175388	0.292968633
H2a	1.792992326	2.992955277	-1.462936435
H2e	1.109141932	3.115674967	-3.060298442
H1a	1.381055226	0.712882917	-3.441961895
H1e	2.907989815	1.484684952	-3.079728364
H1am	4.108466101	0.981975593	-1.214168854
H2am	3.525668364	-0.217795171	-0.053603393
H1m	3.002753531	4.610811471	1.125198179
H1m	3.995893789	3.800622546	2.355710102
H1m	2.282667694	3.368551192	2.169838923
O1	-1.609592829	1.618076523	0.622414449
N1	-0.114317087	2.130888124	-1.62440243
N2	-2.549440281	3.683899844	0.517184859

C1	-2.343345099	1.199280809	-2.329199896
C2	-1.07556594	1.922737839	-2.734520777
C3	-0.549357699	3.239662324	-0.762647723
C4	-1.636202188	2.790053467	0.183026511
C5	-3.664839381	3.462851312	1.429556993
H1n	-2.495495373	4.583719595	0.063210439
H2a	-2.913615606	1.785248231	-1.613874383
H2e	-2.980804614	1.096251587	-3.214334825
H1a	-0.565927866	1.355490032	-3.510073046
H1e	-1.343067046	2.886005143	-3.182258806
H1am	-0.862752679	4.111294384	-1.344966027
H2am	0.28735564	3.539167901	-0.131935976
H1m	-4.594325909	3.387020344	0.862904029
H1m	-3.734153101	4.304228177	2.11697783
H1m	-3.51766933	2.544438152	1.986758274
O1	-1.646977359	-1.509292469	0.570266037
N1	-2.060448608	-0.1221979	-1.730076669
N2	-3.631706488	-2.571582727	0.373009999
C1	-1.071621257	-2.357337026	-2.32059648
C2	-1.75845811	-1.095842589	-2.805718727
C3	-3.217939568	-0.576302721	-0.945342155
C4	-2.781560879	-1.610269381	0.063633629
C5	-3.369919963	-3.595831482	1.372357639
H1n	-4.513111203	-2.595692627	-0.11681614
H2a	-1.705335834	-2.905517401	-1.629070842
H2e	-0.910911059	-3.020317058	-3.176911296
H1a	-1.12562295	-0.598837139	-3.536372738
H1e	-2.679449697	-1.372630555	-3.330384696
H1am	-4.02670936	-0.946931177	-1.582005997
H2am	-3.606598497	0.261921967	-0.367792181
H1m	-3.207932493	-4.56092378	0.890144576
H1m	-4.221194704	-3.670263025	2.047279075
H1m	-2.47953384	-3.319202871	1.926613853
O1	1.584489731	-1.57799435	0.667705273
N1	0.213044093	-2.058462918	-1.647769494
N2	2.630019245	-3.574832029	0.483134287
C1	2.48725988	-1.103862495	-2.179913696
C2	1.254516873	-1.832204227	-2.680701326
C3	0.58663803	-3.180248454	-0.767627438
C4	1.653872985	-2.731172168	0.197386542
C5	3.723105776	-3.287868326	1.399325171
H1n	2.613413192	-4.47843504	0.035705746
H2a	3.022091088	-1.698582947	-1.446256473
H2e	3.175176652	-0.970986404	-3.021446936
H1a	0.801129491	-1.25899179	-3.486288229
H1e	1.56089043	-2.789031258	-3.11695412
H1am	0.894779064	-4.060827635	-1.339315944
H2am	-0.271865228	-3.445447517	-0.151663929
H1m	4.673936865	-3.391103842	0.876650095
H1m	3.701146659	-3.977127153	2.242668867
H1m	3.618555375	-2.271285001	1.764686793
Ow	0.034252734	-3.823695508	2.478818106
Hw	0.021804526	-4.705338358	2.870929171
Hw	-0.482596839	-3.268231685	3.09755156
Ow	2.538219322	0.156703563	3.210337413
Hw	1.711374924	0.137984861	2.683845815
Hw	2.383272632	0.838091877	3.876604745
Ow	1.967503767	-2.367297363	4.169196778

Hw	1.583208227	-2.850718751	3.417663421
Hw	2.238712406	-1.489754254	3.822141474
Ow	-0.305782329	1.773305189	3.944201683
Hw	-0.574355644	2.618126839	3.565532923
Hw	-0.169432536	1.158143773	3.176198894
Ow	-0.798912147	-2.280379766	4.66029691
Hw	0.153526018	-2.073778009	4.662951985
Hw	-1.290538549	-1.437953262	4.625729235
Ow	-2.28796343	0.12279147	4.812596793
Hw	-2.718938237	0.34267079	5.647390753
Hw	-1.630117973	0.835716664	4.634740289
Ow	-3.574122166	0.270264188	2.336111025
Hw	-2.801955897	0.573143321	1.83760845
Hw	-3.215957211	0.111875482	3.229998661

7 References

[1] S.Aime,A. Barge, J.I. Bruce, M. Botta, J.A.K. Howard, J.M. Moloney, D. Parker, A.S. de Sousa, M. Woods, *J. Am. Chem. Soc.* **1999**, *121*, 5762–5771.
[2] P. Kuzmic, *Anal. Biochem.* **1996**, *237*, 260–273.
[3] Electron Paramagnetic Resonance; A. Abragam, B. Bleaney, Eds.; Oxford: Clarendon Press: Oxford, 1970.
[4] S. Stoll, A. Schweiger, *J. Magn. Reson.* **2006**, *178*, 42–55.
[5] R.G. Denning, J. Harmer, J.C. Green, M. Irwin, *J. Am. Chem. Soc.* **2011**, 133, 20644-20660.
[6] (a) C. Lee, W. Yang, R.G. Parr, *Phys. Rev. B* **1988**, *37*, 785. (b) A.D. Becke. *Phys. Rev. A* **1988**, *38*, 3098. (c) A.D. Becke, *J. Chem. Phys.* **1993**, *98*, 5648.
[7] (a) S. Grimme, J. Antony, S. Ehrlich, H. Krieg, H. *J. Chem. Phys.* **2010**, *132*, 154104. (b) S. Grimme, S. Ehrlich, L. Goerigk, *J. Comput. Chem.* **2011**, *32*, 1456.
[8] (a) F. Neese, F. Wennmohs, A. Hansen, U. Becker, *Chem. Phys.* **2009**, *356*, 98. (b) R. Izsák, F. Neese, *J. Chem. Phys.* **2011**, *135*, 144105.
[9] (a) E. van Lenthe, E. E.J. Baerends, J.G. Snijders, *J. Chem. Phys.* **1993**, *99*, 4597. (b) C. van Wüllen, C. *J. Chem. Phys.* **1998**, *109*, 392.
[10] (a) A. Schäfer, H. Horn, R. Ahlrichs, *J. Chem. Phys.* **1992**, *97*, 2571. (b) F. Weigend, R. Ahlrichs, *Phys. Chem. Chem. Phys.* **2005**, *7*, 3297. (c) D.A. Pantazis, F. Neese, *J. Chem. Theory Comput.* **2009**, *5*, 2229.
[11] F. Neese, *Wiley Interdiscip. Rev. Comput. Mol. Sci.* **2012**, *2*, 73.
[12] (a) L. Ungur, L.F. Chibotaru, *Phys. Chem. Chem. Phys.* **2011**, *13*, 20086. (b) R.J. Blagg, L. Ungur, F. Tuna, J. Speak, P. Comar, D. Collison, W. Wernsdorfer, E.J.L. McInnes, L.F. Chibotaru, R.E.P. Winpenny, *Nat. Chem.* **2013**, *5*, 673. (c) L. Ungur, J.J. Le Roy, I. Korobkov, M. Murugesu, L.F. Chibotaru, *Angew. Chem. Int. Ed.* **2014**, *53*, 4413.
[13] (a) G. Karlström, R. Lindh, P.A. Malmqvist, B.O. Roos, U. Ryde, V. Veryazov, P.O. Widmark, M.Cossi, B. Schimmelpfennig, P. Neogrady, L. Seijo, *Comput. Mater. Sci.* **2003**, *28*, 222. (b) V. Veryazov, P. Widmark, L. Serrano-Andrés, R. Lindh, B.O. Roos, *Int. J. Quantum Chem.* **2004**, *100*, 626. (c) F. Aquilante, L. De Vico, N. Ferré, G. Ghigo, P. Malmqvist, P. Neogrády, T.B. Pedersen, M. Pitoňák, M. Reiher, B.O. Roos, L. Serrano-Andrés, M. Urban, V. Veryazov, R. Lindh, *J. Comput. Chem.* **2010**, *31*, 224.
[14] (a) B.O. Roos, V. Veryazov, P.O. Widmark, *Theor. Chem. Acc.* **2004**, *111*, 345.

(b) B.O. Roos, R. Lindh, P.Å Malmqvist,.; V. Veryazov, P.O. Widmark, *J. Phys. Chem. A* **2004**, *108*, 2851.
(c) B.O. Roos, R. Lindh, P.Å Malmqvist, V. Veryazov, P.O. Widmark, *J. Phys. Chem. A* **2005**, *109*, 6575.
(d) B.O. Roos, R. Lindh, P.Å Malmqvist, V. Veryazov, P.O. Widmark *Chem. Phys. Lett.* **2005**, *409*, 295.
[15] N. F. Chilton, R. P. Anderson, L. D. Turner, A. Soncini, K. S. Murray, *J. Comput. Chem.*, **2013**, 34, 1164.
